# Comparative efficacy and pharmacological mechanism of Chinese patent medicines against anthracycline-induced cardiotoxicity: An integrated study of network meta-analysis and network pharmacology approach

**DOI:** 10.3389/fcvm.2023.1126110

**Published:** 2023-04-24

**Authors:** Yifei Rao, Yu Wang, Zhijian Lin, Xiaomeng Zhang, Xueli Ding, Ying Yang, Zeyu Liu, Bing Zhang

**Affiliations:** ^1^ School of Chinese Materia Medica, Beijing University of Chinese Medicine, Beijing, China; ^2^ Center for Pharmacovigilance and Rational Use of Chinese Medicine, Beijing University of Chinese Medicine, Beijing, China

**Keywords:** anthracycline-induced cardiotoxicity, Chinese patent medicines, network meta-analysis, network pharmacology, comparative efficacy, pharmacological mechanism

## Abstract

**Background:**

This study aimed to evaluate the efficacy of Chinese patent medicines (CPMs) combined with dexrazoxane (DEX) against anthracycline-induced cardiotoxicity (AIC) and further explore their pharmacological mechanism by integrating the network meta-analysis (NMA) and network pharmacology approach.

**Methods:**

We searched for clinical trials on the efficacy of DEX + CPMs for AIC until March 10, 2023 (Database: PubMed, Embase, Cochrane Library, Chinese National Knowledge Infrastructure, China Science and Technology Journal and China Online Journals). The evaluating outcomes were cardiac troponin I (cTnI) level, creatine kinase MB (CK-MB) level, left ventricular ejection fraction (LVEF) value, and electrocardiogram (ECG) abnormal rate. Subsequently, the results of NMA were further analyzed in combination with network pharmacology.

**Results:**

We included 14 randomized controlled trials (RCTs) and 1 retrospective cohort study (*n* = 1,214), containing six CPMs: Wenxinkeli (WXKL), Cinobufotalin injection (CI), Shenqifuzheng injection (SQFZ), Shenmai injection (SM), Astragalus injection (AI) and AI + CI. The NMA was implemented in Stata (16.0) using the mvmeta package. Compared with using DEX only, DEX + SM displayed the best effective for lowering cTnI level (MD = −0.44, 95%CI [−0.56, −0.33], SUCRA 93.4%) and improving LVEF value (MD = 14.64, 95%CI [9.36, 19.91], SUCRA 98.4%). DEX + SQFZ showed the most effectiveness for lowering CK-MB level (MD = −11.57, 95%CI [−15.79, −7.35], SUCRA 97.3%). And DEX + AI + CI has the highest effectiveness for alleviating ECG abnormalities (MD = −2.51, 95%CI [−4.06, −0.96], SUCRA 96.8%). So that we recommended SM + DEX, SQFZ + DEX, and DEX + AI + CI as the top three effective interventions against AIC. Then, we explored their pharmacological mechanism respectively. The CPMs' active components and AIC-related targets were screened to construct the component-target network. The potential pathways related to CPMs against AIC were determined by KEGG. For SM, we identified 118 co-targeted genes of active components and AIC, which were significantly enriched in pathways of cancer pathways, EGFR tyrosine kinase inhibitor resistance and AGE-RAGE signaling pathway in diabetic complications. For SQFZ, 41 co-targeted genes involving pathways of microRNAs in cancer, Rap1 signaling pathway, MAPK signaling pathway, and lipid and atherosclerosis. As for AI + CI, 224 co-targeted genes were obtained, and KEGG analysis showed that the calcium signaling pathway plays an important role except for the consistent pathways of SM and SQFZ in anti-AIC.

**Conclusions:**

DEX + CPMs might be positive efficacious interventions from which patients with AIC will derive benefits. DEX + SM, DEX + SQFZ, and DEX + AI + CI might be the preferred intervention for improving LVEF value, CK-MB level, and ECG abnormalities, respectively. And these CPMs play different advantages in alleviating AIC by targeting multiple biological processes.

## 1. Introduction

Anthracyclines, representative drugs such as doxorubicin, daunorubicin, and epirubicin, are the cornerstone in treating various cancers. However, dose-dependent cardiotoxicity always significantly limited the clinical application of anthracyclines (1). Research reported that cumulative doxorubicin doses of 400, 550, and 700 mg/m^2^ could increase the incidence of congestive heart failure by 4.7%, 26%, and 48% (2). The cumulative toxic effect on the heart will affect oncology patients' prognosis of seriously (3). Thus, the treatment and prevention of AIC is a significant challenge and cannot be ignored. So far, dexrazoxane (DEX) remains the only drug that works as a cardioprotective agent against AIC, which has been approved by the U.S. Food and Drug Administration (4, 5). But DEX inevitably induces adverse effects, such as myelosuppression and secondary malignancies (6). Hence, optimizing the clinical treatment of AIC to improve patient's quality of life deserves further exploration.

Several scholars have tried to explore some profitable ways against AIC from the perspective of the combination of traditional Chinese medicine (TCM) and western medicine. The concept of integrated TCM and western medicine originated in the 17th century (7). Long-term historical experiences show that the cooperation of TCM and western medicine may be more efficient for the cure and prevention of disease than each of them separately. Numerous studies reported the potential benefits of combined TCM and western medicine in improving tumor prognosis, reducing adverse reactions, and enhancing life quality (8–12). Recent clinical evidence indicates that the cooperation of Chinese patent medicines (CPMs) and DEX had a good effect on lowing cardiotoxicity and improving the anti-tumor efficiency of anthracyclines (13). There are kinds of CPMs commonly selected, such as Wenxinkeli (WXKL), Cinobufotalin injection (CI), Shenqifuzheng injection (SQFZ), Shenmai injection (SM), Astragalus injection (AI) and so on. However, the comparative efficacy of these CPMs in treating AIC remains unknown. And their clinical improvement characteristics, active components, and potential targets still need to be well clarified.

Therefore, for comprehensive information for clinicians to determine the optimal combination of CPMs and DEX for patients with AIC, a network meta-analysis was performed in the present study to evaluate the efficacy of different CPMs and rank their effectiveness. All of these CPMs are authorized by the China State Food and Drug Administration. At the same time, network pharmacology will further analyze the more effective CMPs with a high evidence grade to explore their anti-AIC mechanism.

## 2. Methods

### 2.1. The network meta-analysis of the combination of CPMs and DEX against AIC

#### 2.1.1. Protocol registration and search strategies

This study protocol has been registered in the international prospective register of systematic reviews (PROSPERO) (https://www.crd.york.ac.uk/PROSPERO) (14). The registration number is CRD42022297523. We searched PubMed, Embase, Cochrane Library, Chinese National Knowledge Infrastructure (CNKI), China Science and Technology Journal (CSTJ) and China Online Journals (COJ) from inception to March 10, 2023. The initial search items were “anthracycline”, “cardiotoxicity” (title/abstract) and “randomized controlled trial” (title/abstract). The search was conducted using a combination of medical subject headings (MeSH) and free-text words, and the detailed search terms were listed in Supplementary Table S1.

#### 2.1.2. Eligibility criteria

The inclusion criteria were: (1) Clinical trials related to AIC. (2) Patients: All included patients were diagnosed with tumors and received anthracyclines, such as epirubicin, pirarubicin, or doxorubicin. The dose and duration of drug treatment and the gender and age of patients were unlimited. (3) Interventions: before chemotherapy, the experiment group used the combination of CPMs and DEX to counteract the AIC, and the control groups only used DEX. (4) Outcomes: cardiac troponin I (cTnI), creatine kinase MB (CK-MB) or left ventricular ejection fraction (LVEF) at baseline and after chemotherapy, and the rate of abnormal electrocardiogram (ECG). (5) The language of the literature is not limited.

The exclusion criteria were: (1) Repetitive published studies. (2) Non-clinical trials. (3) Cardiotoxicity due to non-anthracycline chemotherapy. (4) Studies with incomplete or incorrect data. (5) Animal experiments, literature review, and conference abstracts.

#### 2.1.3. Data extraction

Two investigators assessed all trials for eligibility and extracted data by screening the titles and abstracts independently. If disagreements occurred, the third investigator judged by retrieving the full articles and discussion. Data extracted included: (1) Basic information, including title, source, author, and year. (2) Baseline characteristics of the study population, containing the number of trial participants, age, and disease type. (3) Intervention details and follow-up time. (4) Key elements of the risk of bias evaluation. (5) Data on outcome indicators and outcome measures of cnTI, CK-MB or LVEF at baseline and after chemotherapy, and the rate of abnormal ECG.

#### 2.1.4. Quality assessment

We considered the following aspects for quality assessment: random sequence generation, allocation concealment, blinding, incomplete outcome data, selective reporting, and other biases. Quality assessment was performed using Review Manager (version 5.3; The Cochrane Collaboration, London, United Kingdom). Disagreements between the two investigators were resolved by consensus.

#### 2.1.5. Statistical analysis

We applied Stata 16 software (StataCorp, College Station, TX, United States) to perform the network meta-analyses. The normalized mean difference (MD) was calculated for continuous variables, the odds ratios (ORs) were used as the effect analysis statistic for dichotomous variables. All results were provided with its 95% confidence interval (95%CI), *I*^2^ statistics and chi-square tests were used to assess statistical heterogeneity. Fixed effects were used if there was no heterogeneity between studies (*I*^2^ < 50%, *P* > 0.1). If there was heterogeneity (*I*^2^ > 50%, *P* < 0.1), the source of heterogeneity was analyzed, and a meta-analysis was performed with random effects after excluding the influence of heterogeneity.

The mvmeta package was used for preprocessing data and drawing the network relationship of different intervention measures. Network meta-analysis (multiple treatments meta-analysis, mixed treatment comparisons) allows the comparison of any two treatments within the network even a direct comparison from a trial is not available.

Such as combine the information from direct evidence (pairwise comparisons) and indirect evidence (comparing B–C from comparisons of A–B and A–C). The outcome indicators for each intervention were ranked by plotting the surface under the cumulative ranking curve (SUCRA), which expressed a percentage of the efficacy or safety of every intervention relative to an imaginary intervention (15). A larger SUCRA score was considered to indicate a more effective or safer intervention. Comparison-adjusted funnel plots were used to assess publication bias and the effects of the small sample in included studies.

### 2.2. The network pharmacology analysis of CPMs against AIC

#### 2.2.1. Acquisition of AIC-related targets

AIC-related targets were obtained from the Genecards database (https://www.genecards.org/) and the DisGeNET database (http://www.disgenet.org/) (16). The search phrases contained “anthracycline-induced cardiotoxicity”, “anthracycline-induced heart failure”, “anthracycline-induced cardiomyopathy”, “adriamycin-induced heart failure”, “doxorubicin-induced cardiotoxicity”, and “doxorubicin-induced cardiomyopathy”. The database of AIC-related targets was established after removing repetitions.

#### 2.2.2. Screen of CPMs-related targets

The basic information of the effective components and compounds of CMPs were obtained through various literature search. The related targets of CPMs components were obtained from the Traditional Chinese Medicine Systems Pharmacology Database and Analysis Platform (TCMSP, http://tcmspw.com/tcmsp.php) and the SwissTargetPrediction database (STP, http://www.swisstargetprediction.ch/) (17, 18). The canonical SMILES information of each ingredient was obtained from PubChem (https://pubchem.ncbi.nlm.nih.gov/) and was imported into the STP to predict the potential targets (19). Only the targets with probability >0.11 were retained. Then, the Uniprot Knowledgebase (http://www.uniprot.org/) was used for gene standardization (20). The overlapping part of the AIC-related targets and CPMs-related targets are the targets of this article that we researched. VENNY2.1 (https://bioinfogp.cnb.csic.es/tools/venny/) was used to obtain the Venn diagram and their intersection targets (21).

#### 2.2.3. Construction of protein-protein interaction network

To further identify the core regulatory targets, The protein-protein interaction (PPI) network was performed by submitting the overlapping targets of active ingredients to the STRING database (https://string-db.org/) (22), which is used for searching known and predicted interactions between proteins. The species were selected as “Homo sapiens”, and score >0.4 was set as statistically significant. The obtained protein interaction relationship results were exported in TSV format, and the acquired data were imported into Cytoscape 3.8.2 (23). Then, the topological analysis was performed, and targets with a degree value greater than the median were selected as the high correlation targets for further research.

#### 2.3.4. Analysis of functional enrichment

To clarify the role of core targets in gene function, Kyoto Encyclopedia of Genes and Genomes (KEGG) enrichment analysis were performed *via* R packages (24). Bubbles and histograms were used to visualize the result. Pathways with adjusted *P *< 0.01 were considered statistically significant. The terms were selected for visualization according to the *P*-value.

## 3. Results

### 3.1. The network meta-analysis of the combination of CPMs and DEX against AIC

**Figure 1:**
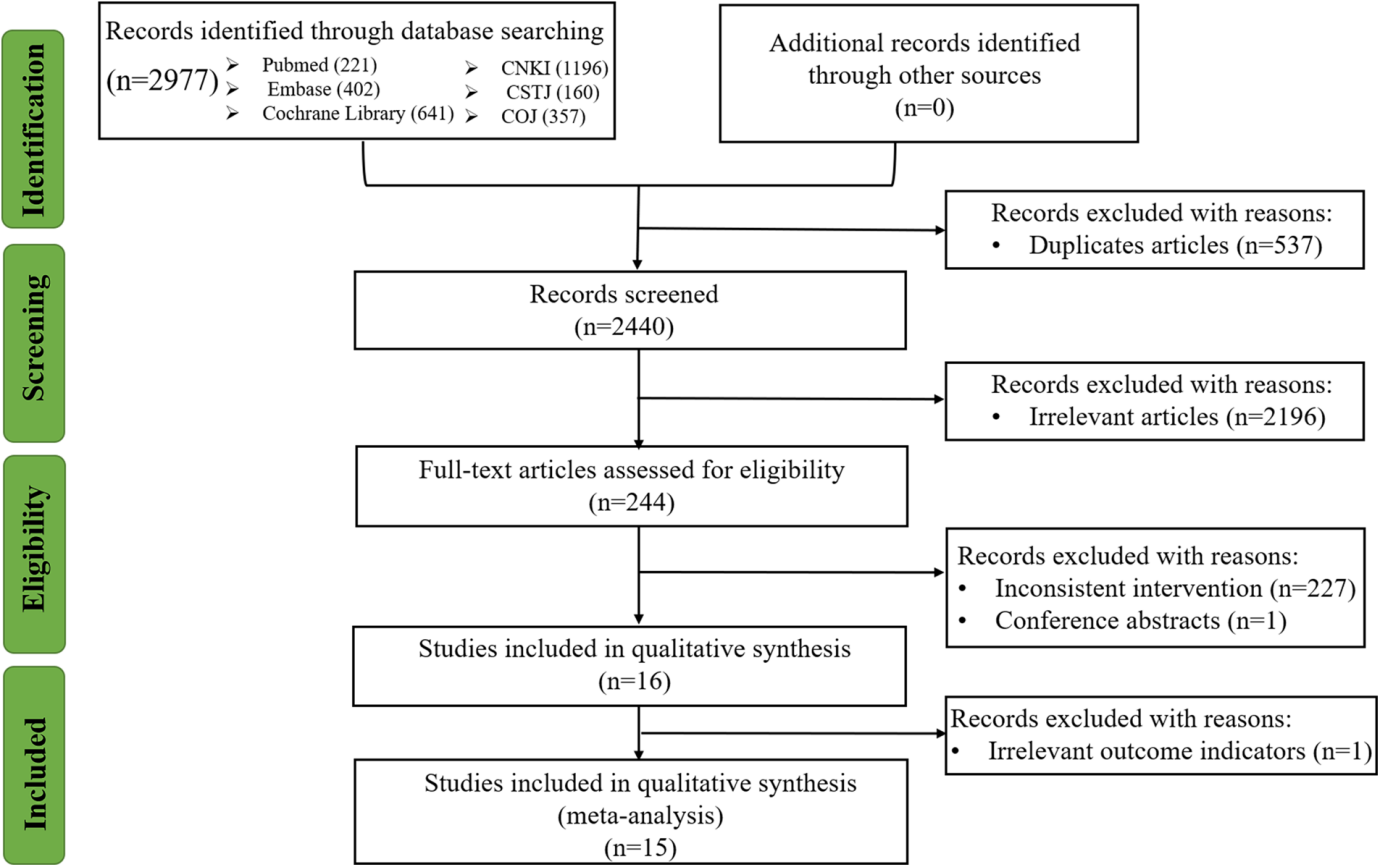
Flow chart of study selection.

We searched a total of 2,977 articles. After the movement of 537 duplicate references and the deletion of 2,196 irrelevant studies, the full text of the remaining 244 references was scanned. Then, we excluded 227 studies with inconsistent intervention, 1 conference abstract, and 1 literature without qualitative outcomes. Finally, the remaining 15 published studies comprised 14 RCTs and 1 retrospective cohort study, including 1,214 participants and 7 therapy regimens, were available for the network meta-analysis (25–39). The study selection flow chart is shown in Figure 1.

#### 3.1.1. Study characteristics

We selected DEX as the control to compare the effects of different DEX + CPMs interventions on AIC. Six DEX + CPMs interventions include DEX + WXKL, DEX + CI, DEX + SQFZ, DEX + SM, DEX + AI, DEX + AI + CI. Among included 15 RCTs, 12 studies reported the improvement of LVEF value, 8 studies reported a reduction in cTnI level, 7 studies reported a decrease in CK-MB level and 11 studies reported the rate of abnormal ECG in patients. Levels of cTnI and CK-MB as early predictive markers for AIC, which could significantly increase after cardiac injury and reflect the sensitive change of abnormal myocardial status (40). LVEF is an indicator for monitoring cardiac function, the LVEF value with a drop of 10% from the baseline to an absolute value of <50% is commonly used in identifying ACT (41). ECG is also widely used as an initial screening tool for AIC (42). ECG can display a variety of non-specific abnormalities, covering ST-T segment elevation, sinus tachycardia, atrioventricular block and malignant premature beat (43). The comparative efficacy of these DEX + CPMs in treating AIC was performed by network meta-analysis. Detailed characteristics of all included studies are shown in Table 1. The information of main CMPs were shown in the Supplementary Table S2.

**Table 1:** General characteristics in a network meta-analysis of the therapy of anthracycline-induced cardiotoxicity patients.

Author year	State	Mean age		Sample size		Drug type	Tumor diagnosis	Doses	Follow-up duration	Outcome indicators
		T	C	T	C					
Renjianlin et al., 2020 (25)	China	53.1 ± 7.0	52.2 ± 6.5	50	50	Wenxinkeli	Breast cancer	NA	84, day	LVEF, rate of abnormal ECG
Wangsiyuan et al., 2020 (26)	China	60–75	60–75	60	60	Shenqifuzheng injection	Malignant tumor	250 ml	60, day	LVEF, CK-MB
Jiangzhuhui et al., 2018 (27)	China	44.6 ± 8.7	44.3 ± 9.9	42	38	Astragalus injection	Hematological tumor	10–20/250 ml	40, day	cTnI, CK-MB, rate of abnormal ECG
Lvzhenhui et al., 2017 (28)	China	36.3 ± 5.3	38.2 ± 5.5	28	28	Shenmai injection	Acute leukemia	40 ml	14, day	LVEF, CK-MB, rate of abnormal ECG
Wangxuan et al., 2017 (29)	China	18–78	18–78	30	30	Shenmai injection	Hematological tumor	50 mg	NA	cTnI, rate of abnormal ECG
Chenshuzhi et al., 2017 (30)	China	20–72	20–72	30	30	Shenqifuzheng injection	Malignant tumor	250 ml	28, day	LVEF, cTnI, rate of abnormal ECG
Linwenbo, 2016 (31)	China	38.9 ± 5.8	39.2 ± 5.6	32	32	Shenmai injection	Malignant tumor	50 mg	7, day	LVEF, CK-MB
Zhangzhenjiang, 2016 (32)	China	51.8 ± 3.3	51.7 ± 3.4	25	25	Shenmai injection	Acute leukemia	50 mg	NA	LVEF, cTnI, rate of abnormal ECG
Zhangguowen, 2016 (33)	China	48.3 ± 5.4	47.6 ± 4.9	75	61	Cinobufotalin injection	Malignant tumor	15/500 ml	28, day	LVEF, cTnI, CK-MB, rate of abnormal ECG
Yuwei et al., 2015 (34)	China	18–65	18–65	30	30	Shenmai injection	Breast cancer	50 ml	84, day	LVEF, rate of abnormal ECG
Wangxuan, 2015 (35)	China	18–78	18–78	35	35	Shenmai injection	acute leukemia	50 mg	NA	cTnI, rate of abnormal ECG
Luorenfeng et al., 2015 (36)	China	33–69	33–69	30	30	Astragalus injection	Malignant tumor	20/250 ml	28, day	LVEF, cTnI, CK-MB, rate of abnormal ECG
Luorenfeng et al., 2015 (36)	China	33–69	33–69	30	30	Cinobufotalin injection	Malignant tumor	20/250 ml	28, day	LVEF, cTnI, CK-MB, rate of abnormal ECG
Luorenfeng et al., 2015 (36)	China	33–69	33–69	30	30	Astragalus with cinobufotalin injection	Malignant tumor	40/500 ml	28, day	LVEF, cTnI, CK-MB, rate of abnormal ECG
Fengqiong, 2014 (37)	China	49.4 ± 5.7	51.2 ± 5.6	32	28	Shenqifuzheng injection	Breast cancer	250 ml	126, day	LVEF
Wangxuan, 2014 (38)	China	28–75	28–75	30	30	Shenmai injection	Malignant tumor	50 mg	12, day	LVEF, CK-MB
Zhuanghaifeng et al., 2012 (39)	China	17–78	17–78	30	30	Shenmai injection	Hematological tumor	50 ml	14, day	LVEF, cTnI, rate of abnormal ECG

NA, data not available; T, treatment group; C, control group; d, days; LVEF, left ventricular ejection fraction; cTnI, cardiac troponins I; CK-MB, creatine kinase—MB; ECG, electrocardiogra.

#### 3.1.2. Quality assessment

**Figure 2:**
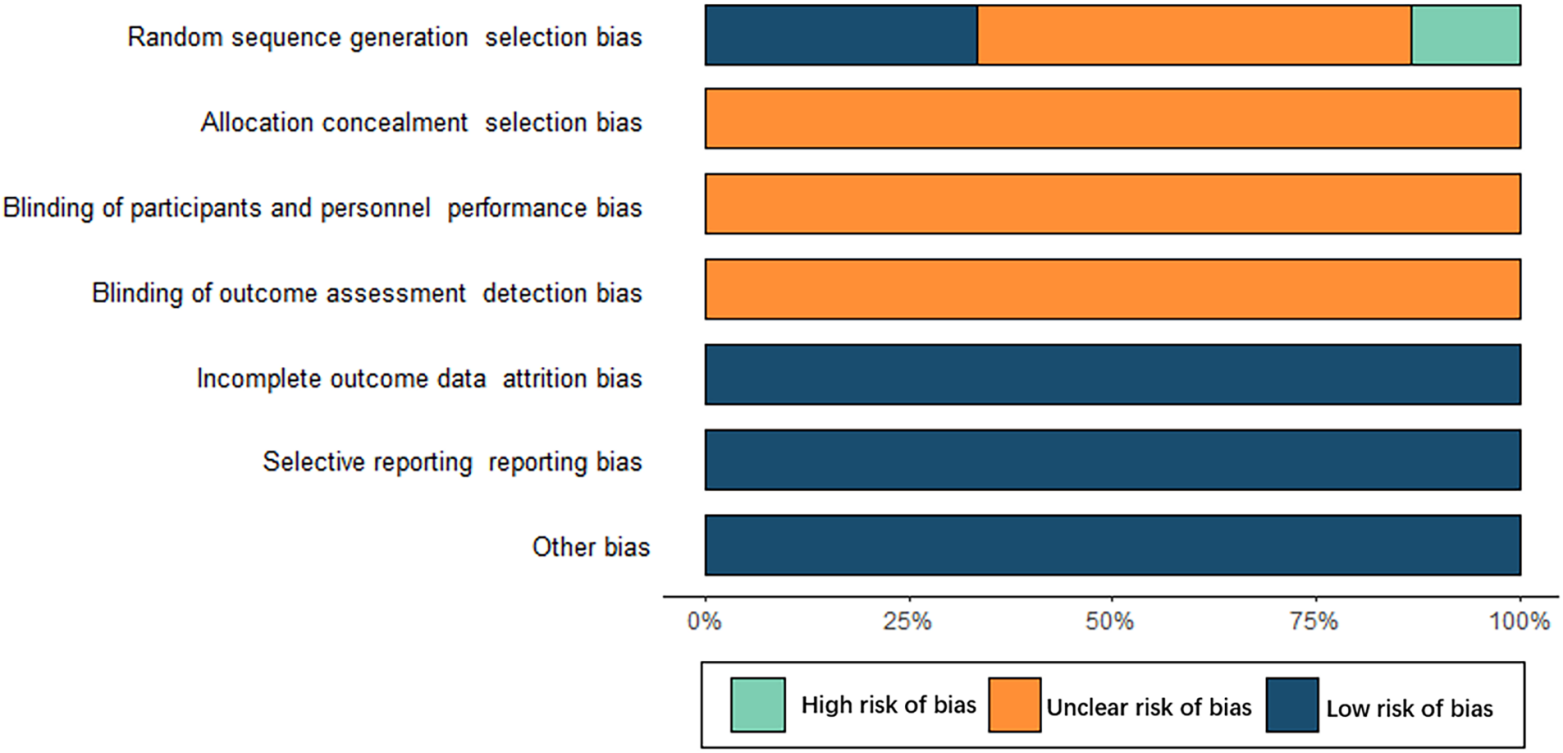
Results of risk of bias assessment.

Two investigators independently assessed the risk of bias in the included studies and cross-checked the results. Data were collated from 15 published articles comprising 14 RCTs and 1 retrospective cohort study. 14 RCTs were all assigned in random group. Concretely, 5 RCTs performed the “random number table” method, 1 RCT used the “treatment order” method, 1 RCT employed the “medication method”, and the rest studies failed to specify the randomization method. All studies did not report allocation concealment. Blinding was assessed as an unclear source of bias because of insufficient information. Regarding data completeness, selective reporting and other aspects showed a low risk of bias. The results of a detailed bias evaluation are shown in Figure 2.

#### 3.1.3. Outcome of cTnI level

**Figure 3:**
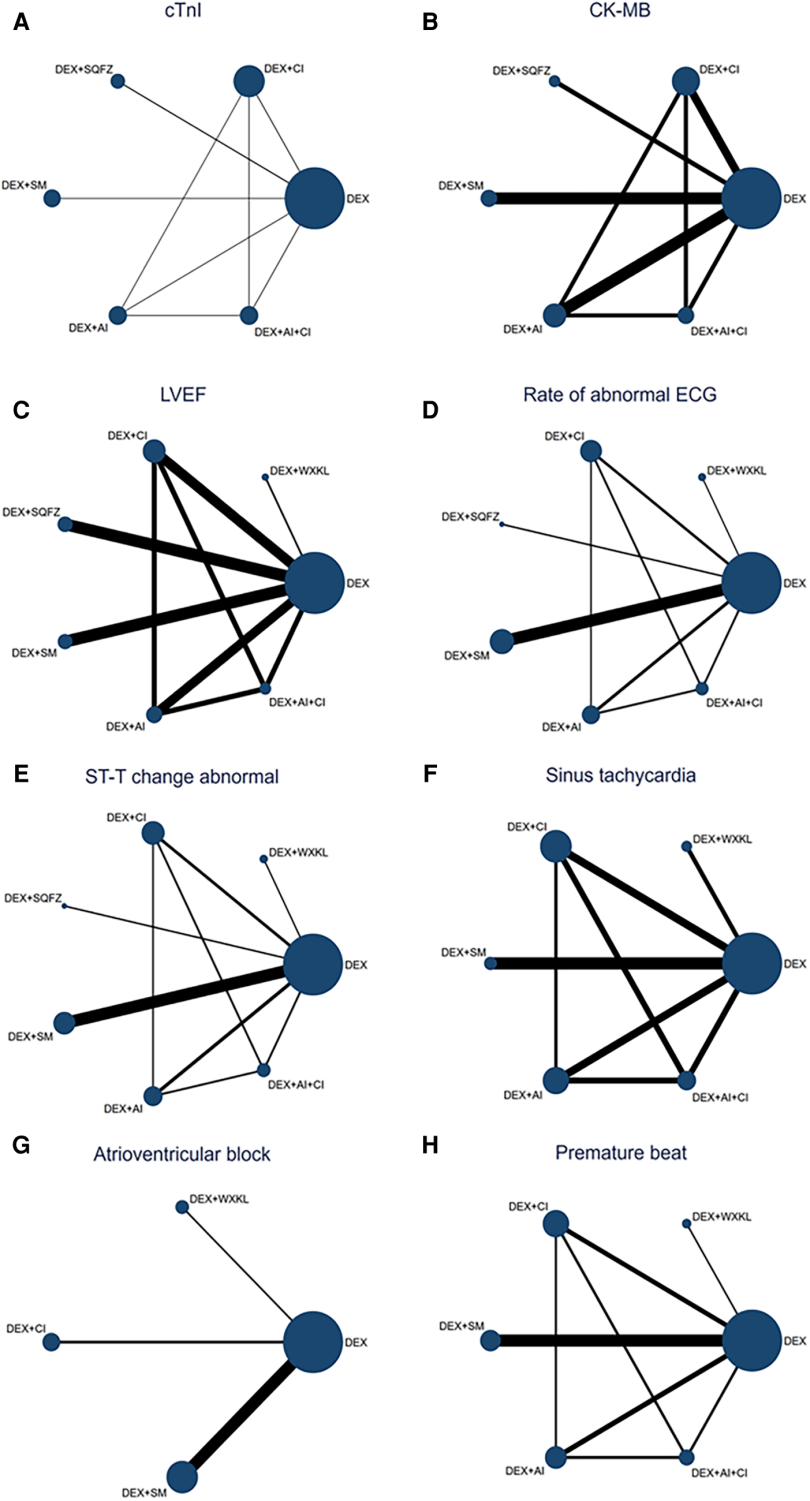
Network map for all outcomes. (**A**) cTnI. (**B**) CK-MB. (**C**) LVEF. (**D**) Rate of abnormal of ECG. (**E**) ST-T change abnormal. (**F**) Sinus tachycardia. (**G**) Atrioventricular block. (**H**) Premature beat. (DEX, Dexrazoxane; DEX + WXKL, Dexrazoxane combined with Wenxinkeli; DEX + CI, Dexrazoxane combined with Cinobufotalin injection; DEX + SQFZ, Dexrazoxane combined with Shenqifuzheng injection; DEX + SM, Dexrazoxane combined with Shenmai injection; DEX + AI, Dexrazoxane combined with Astragalus injection; DEX + AI + CI, Dexrazoxane combined with Astragalus with Cinobufotalin injection). The size of every node is proportional to the number of participants (sample size). Lines represent the available direct comparisons between pairs of treatments. Their width is proportional to the number of trials comparing every pair of treatments.

**Figure 4:**
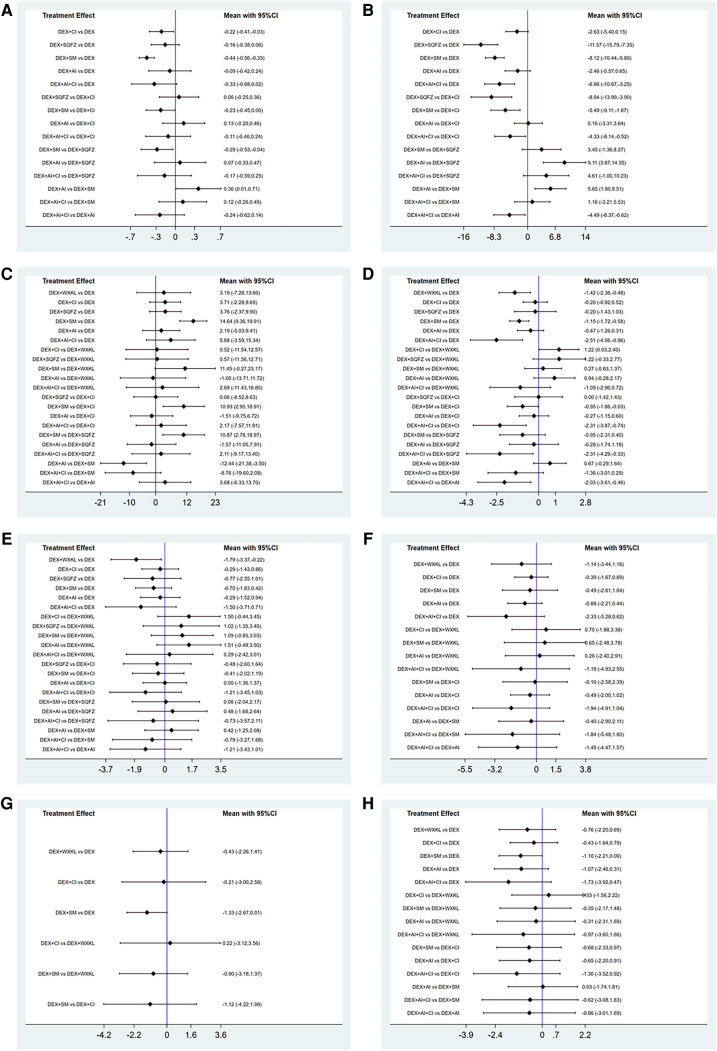
Forest plot of the studies included. (**A**) cTnI. (**B**) CK-MB. (**C**) LVEF. (**D**) Rate of abnormal of ECG. (**E**) ST-T change abnormal. (**F**) Sinus tachycardia. (**G**) Atrioventricular block. (**H**) Premature beat (DEX, Dexrazoxane; DEX + WXKL, Dexrazoxane combined with Wenxinkeli; DEX + CI, Dexrazoxane combined with Cinobufotalin injection; DEX + SQFZ, Dexrazoxane combined with Shenqifuzheng injection; DEX + SM, Dexrazoxane combined with Shenmai injection; DEX + AI, Dexrazoxane combined with Astragalus injection; DEX + AI + CI, Dexrazoxane combined with Astragalus with Cinobufotalin injection).

As shown in Figure 3A, 7 studies containing 5 therapeutic regimens (DEX + CI, DEX + SQFZ, DEX + SM, DEX + AI, DEX + AI + CI) reported the cTnI level. The network meta-analysis results in the cTnI level are displayed in Figure 4A. The cTnI level in different DEX + CPMs treatments were significantly decreased compared with DEX treatment. The detailed comparative statistics were DEX + CI vs. DEX (MD = −0.22, 95% CI [−0.41, −0.03]), DEX + SQFZ vs. DEX (MD = −0.16, 95% CI [−0.38, 0.06]), DEX + SM vs. DEX (MD = −0.44, 95% CI [−0.56, −0.33]), DEX + AI vs. DEX (MD = −0.09, 95% CI [−0.42, 0.24]), DEX + AI + CI vs. DEX (MD = −0.33, 95% CI [−0.68, 0.02]).

**Figure 5:**
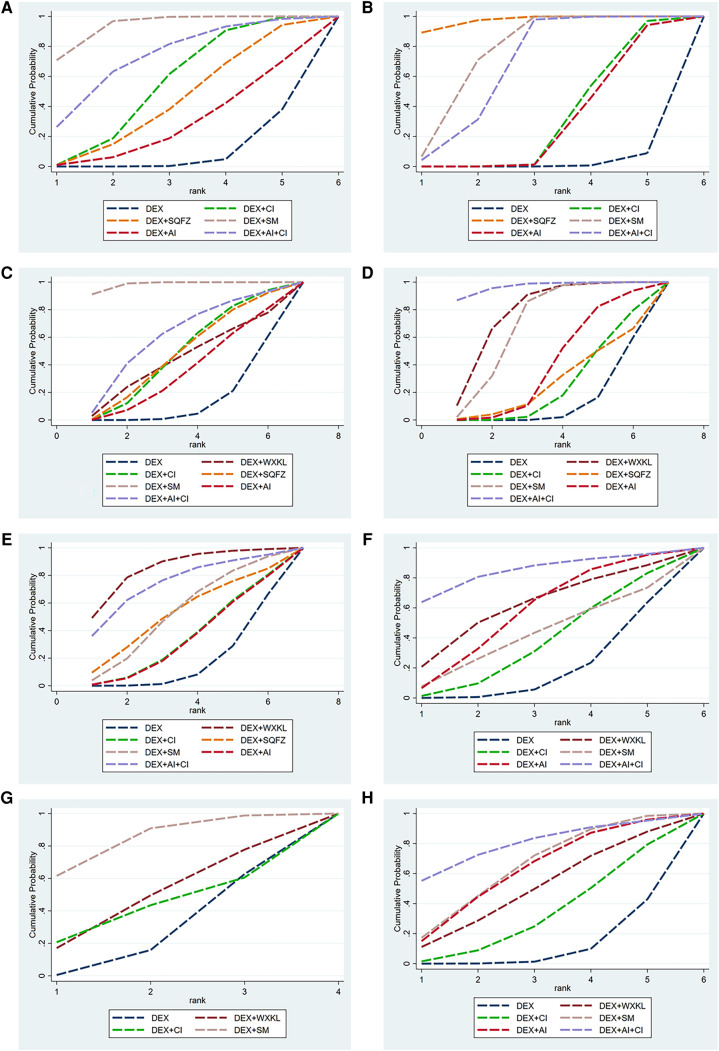
The surface under the cumulative ranking curve (SUCRA) is shown for each treatment. (**A**) cTnI. (**B**) CK-MB. (**C**) LVEF. (**D**) Rate of abnormal of ECG. (**E**) ST-T change abnormal. (**F**) Sinus tachycardia. (**G**) Atrioventricular block. (**H**) Premature beat (DEX, Dexrazoxane; DEX + WXKL, Dexrazoxane combined with Wenxinkeli; DEX + CI, Dexrazoxane combined with Cinobufotalin injection; DEX + SQFZ, Dexrazoxane combined with Shenqifuzheng injection; DEX + SM, Dexrazoxane combined with Shenmai injection; DEX + AI, Dexrazoxane combined with Astragalus injection; DEX + AI + CI, Dexrazoxane combined with Astragalus with Cinobufotalin injection).

**Figure 6:**
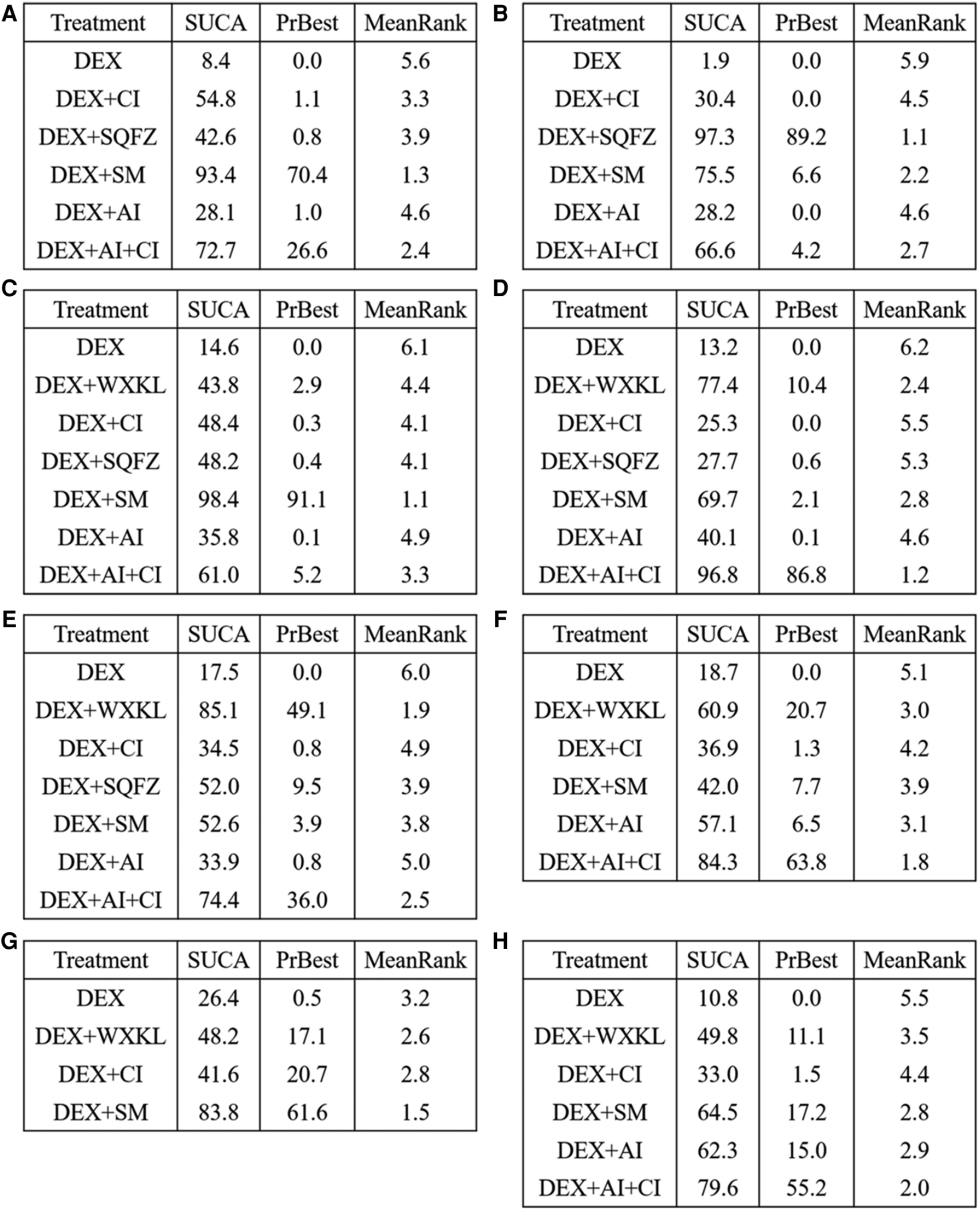
SUCRA rank of each intervention. (**A**) cTnI. (**B**) CK-MB. (**C**) LVEF. (**D**) Rate of abnormal of ECG. (**E**) ST-T change abnormal. (**F**) Sinus tachycardia. (**G**) Atrioventricular block. (**H**) Premature beat (DEX, Dexrazoxane; DEX + WXKL, Dexrazoxane combined with Wenxinkeli; DEX + CI, Dexrazoxane combined with Cinobufotalin injection; DEX + SQFZ, Dexrazoxane combined with Shenqifuz)heng injection; DEX + SM, Dexrazoxane combined with Shenmai injection; DEX + AI, Dexrazoxane combined with Astragalus injection; DEX + AI + CI, Dexrazoxane combined with Astragalus with Cinobufotalin injection).

Among these 5 DEX + CPMs therapeutic regimens, the larger SUCRA indicated the better effect in reducing the cTnI level. According to Figures 5A, 6A, the rank of effective medication regimen was DEX + SM (SUCRA = 93.4%), DEX + AI + CI (SUCRA = 72.7%), DEX + CI (SUCRA = 54.8%), DEX + SQFZ (SUCRA = 42.6%), DEX + AI (SUCRA = 28.1%). These data indicate that the patients treated with DEX + SM had the highest probability of reducing the cTnI level.

#### 3.1.4. Outcome of CK-MB level

From Figure 3B, 7 studies containing 5 therapeutic regimens (DEX + CI, DEX + SQFZ, DEX + SM, DEX + AI, DEX + AI + CI) reported the CK-MB level. The network meta-analysis results in Figure 4B showed that the cTnI level in different DEX + CPMs treatments were lower than in the DEX treatment. The detailed comparative statistics were DEX + CI vs. DEX (MD = −2.63, 95% CI [−5.40, 0.15]), DEX + SQFZ vs. DEX (MD = −11.57, 95% CI [−15.79, −7.35]), DEX + SM vs. DEX (MD = −8.12, 95% CI [−10.44, −5.80]), DEX + AI vs. DEX (MD = −2.46, 95% CI [−5.57, 0.65]), and DEX + AI + CI vs. DEX (MD = −6.96, 95% CI [−10.67, −3.25]).

From the Figures 5B, 6B, SQFZ + DEX was the most effective treatment in reducing the CK-MB level for its SUCRA value of 97.3%, followed by DEX + SM (75.5%), DEX + AI + CI (66.6%), DEX + CI (30.4%), DEX + AI (28.2%), and DEX (1.9%). The above results demonstrate that the patients with DEX + SQFZ treatment had the highest probability of decreasing the CK-MB level.

#### 3.1.5. Outcome of LVEF value

In Figure 3C, 12 studies covering six therapeutic regimens (DEX + CI, DEX + SQFZ, DEX + SM, DEX + AI, DEX + AI + CI, and DEX + WXKL) assessed the change in LVEF value. The network meta-analysis results in Figure 4C showed that DEX + SM administration (MD = 14.64, 95% CI [9.36, 19.91]) could significantly increase the LVEF value more than the DEX treatment. At the same time, the other five therapeutic regimens all had no significant differences in the LVEF value from the DEX treatment. And their detailed comparative statistics were DEX + WXKL vs. DEX (MD = 3.19, 95% CI [−7.28, 13.66]), DEX + CI vs. DEX (MD = 3.71, 95% CI [−2.28, 9.69]), DEX + SQFZ vs. DEX (MD = 3.76, 95% CI [−2.37, 9.90]), DEX + AI vs. DEX (MD = 2.19, 95% CI [−5.03, 9.41]), DEX + AI + CI vs. DEX (MD = 5.88, 95% CI [−3.59, 15.34]).

As shown in Figures 5C, 6C, the highest SUCRA value of DEX + SM treatment (98.4%) meant the effective improvement of LVEF, followed by DEX + AI + CI (61.0%), DEX + CI (48.4%), DEX + SQFZ (48.2%), DEX + WXKL (43.8%), DEX + AI (35.8%), and DEX (14.6%). These data indicate that the patients with DEX + SM treatment had the highest probability of improving LVEF value.

#### 3.1.6. Outcome of the abnormal rate of ECG

As displayed in Figure 3D, 11 studies, including 6 therapeutic regimens (DEX + CI, DEX + SQFZ, DEX + SM, DEX + AI, DEX + AI + CI, and DEX + WXKL), calculated the abnormal rate of ECG. The network meta-analysis results in Figure 4D exhibited that intervention protocols of DEX + WXKL, DEX + CI, DEX + SM, DEX + AI, and DEX + AI + CI all had significant improvement in the abnormal rate of ECG when compared with DEX treatment (MD = −1.42, 95% CI [−2.36, −0.48]; MD = −0.20, 95% CI [−0.92, 0.52]; MD = −1.15, 95% CI [−1.72, −0.58]; MD = −0.47, 95% CI [−1.26, 0.31]; MD = −2.51, 95% CI [−4.06, −0.96], respectively), whereas DEX + SQFZ administration (MD = −0.20, 95% CI [−1.43, 1.03]) showed no apparent alleviation in the abnormal rate of ECG.

According to Figures 5D, 6D, the DEX + AI + CI administration displayed the most effective reduction in the abnormal rate of ECG with a SUCRA value of 96.8%, followed by treatment of DEX + WXKL (77.4%), DEX + SM (69.7%), DEX + AI (40.1%), DEX + SQFZ (27.7%), and DEX + CI (25.3%), and DEX (13.2%). These data imply that the patients with DEX + AI + CI treatment had the highest probability of alleviating the abnormal rate of ECG.

#### 3.1.7. Outcome of ST-T segment alteration rates

From Figure 3E, 10 studies covering 6 therapeutic regimens (DEX + CI, DEX + SQFZ, DEX + SM, DEX + AI, DEX + AI + CI, and DEX + WXKL) compared the ECG ST-T segment alteration rates. The network meta-analysis results in Figure 4E showed that intervention protocols of DEX + WXKL, DEX + CI, DEX + SM, DEX + AI, and DEX + AI + CI all significantly decreased the ST-T segment alteration rates when compared with DEX treatment (MD = −1.79, 95% CI [−3.37, −0.22]; MD = −0.29, 95% CI [−1.43, 0.86]; MD = −0.70, 95% CI [−1.83, 0.42]; (MD = −0.29, 95% CI [−1.52, 0.94]; MD = −1.50, 95% CI [−3.71, 0.71], respectively), whereas DEX + SQFZ administration (MD = −0.77, 95% CI [−2.55, 1.01]) had no obvious effect in reducing the ST-T segment (ECG Change) alteration rates.

As shown in Figures 5E, 6E, the DEX + WXKL administration displayed the most effective reduction in the ST-T segment alteration rates with a SUCRA value of 85.1%, followed by DEX + AI + CI (74.4%), DEX + SM (52.6%), DEX + SQFZ (52.0%), DEX + CI (34.5%), DEX + AI (33.9%), and DEX (17.5%). These data suggest that the patients with DEX + WXKL treatment had the highest probability of reducing the ST-T segment alteration rates.

#### 3.1.8. Outcome of sinus tachycardia

As shown in Figure 3F, 6 studies including 5 therapeutic regimens (DEX + CI, DEX + SM, DEX + AI, DEX + AI + CI, and DEX + WXKL), reported the improvement of sinus tachycardia. The network meta-analysis results in Figure 4F displayed that only three therapeutic regimens of CPMs and DEX showed a potent effect in improving sinus tachycardia than DEX treatment, including DEX + CI (MD = −0.39, 95% CI [−1.67, 0.89]), DEX + AI (MD = −0.88, 95% CI [−2.21, 0.44]), and DEX + AI + CI (MD = −2.33, 95% CI [−5.28, 0.62]). And there were no evident differences in sinus tachycardia improvement observed in WXKL + DEX (MD = −1.14, 95% CI [−3.44, 1.16]), DEX + SM (MD = −0.49, 95% CI [−2.61, 1.64]) when compared with DEX treatment.

The SUCRA in Figures 5F, 6F exhibited that the DEX + AI + CI administration had the most potent in improving sinus tachycardia with a SUCRA value of 84.3%, followed by DEX + WXKL (60.9%), DEX + AI (57.1%), DEX + SM (42.0%), DEX + CI (36.9%) and DEX (18.7%). These data indicate that the patients with DEX + AI + CI treatment had the highest probability of sinus tachycardia improvement.

#### 3.1.9. Outcome of atrioventricular block

From Figure 3G, 6 studies, involving 3 therapeutic regimens (DEX + WXKL, DEX + CI, DEX + SM), reported the improvement of the atrioventricular block. The network meta-analysis results in Figure 4G revealed that only DEX + SM administration (MD = −1.33, 95% CI [−2.67, 0.01]) could more significantly ameliorate atrioventricular block than DEX treatment. And other arms, such as DEX + WXKL (MD = −0.43, 95% CI [−2.26, 1.41]) and DEX + CI (MD = −0.21, 95% CI [−3.00, 2.58]), showed no significant differences in atrioventricular block improvement compared with DEX treatment. At the same time, the SUCRA value of DEX + SM administration (83.8%) also indicates that the patients with DEX + SM treatment had the highest probability of relieving atrioventricular block.

#### 3.1.10. Outcome of premature beat

As shown in Figure 3H, 5 studies covering 5 therapeutic regimens (DEX + CI, DEX + SM, DEX + AI, DEX + AI + CI, and DEX + WXKL) reported the improvement of premature beat. The network meta-analysis results in Figure 4H showed that all five therapeutic regimens could improve the premature beat more than the DEX treatment, including DEX + WXKL (MD = −0.76, 95% CI [−2.20, 0.69]), DEX + CI (MD = −0.43, 95% CI [−1.64, 0.79]), DEX + SM (MD = −1.10, 95% CI [−2.21, 0.00]), DEX + AI (MD = −1.07, 95% CI [−2.46, 0.31]), DEX + AI + CI (MD = −1.73, 95% CI [−3.92, 0.47]).

Additionally, as shown in Figures 5H, 6H, the SUCRA of DEX + AI + CI (79.6%) demonstrates that patients had the highest probability of relieving premature beat with DEX + AI + CI treatment, followed by DEX + SM (64.5%), DEX + AI (62.3%), DEX + WXKL (49.8%), DEX + CI (33.0%), and DEX (10.8%).

#### 3.1.11. Publication bias

**Figure 7:**
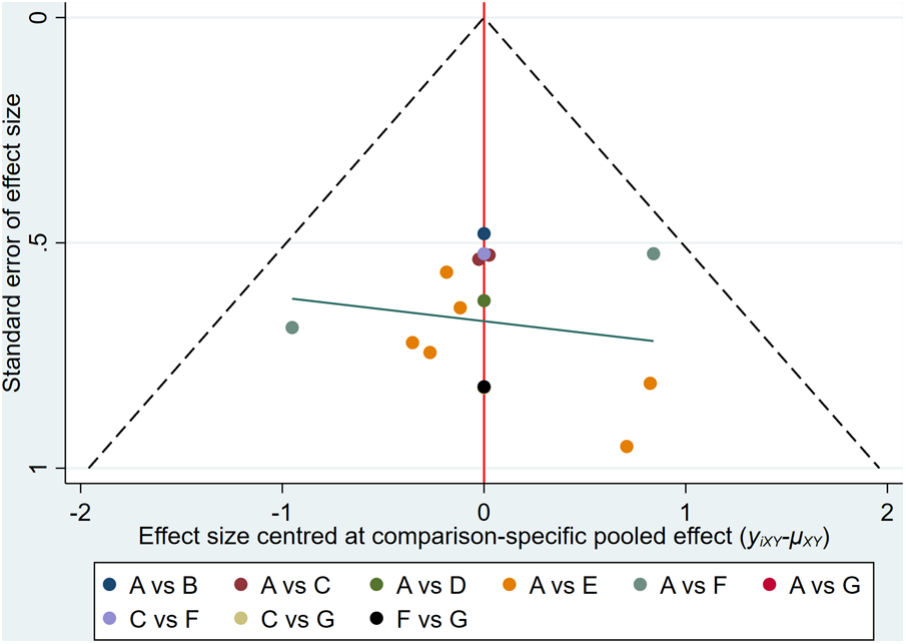
Comparison-correction funnel plot. (A, Dexrazoxane; B, Dexrazoxane combined with Wenxinkeli; C, Dexrazoxane combined with Cinobufotalin injection; D, Dexrazoxane combined with Shenqifuzheng injection; E, Dexrazoxane combined with Shenmai injection; F, Dexrazoxane combined with Astragalus injection; G, Dexrazoxane combined Astragalus with Cinobufotalin injection with).

The abnormal rate of ECG was set as an outcome indicator for publication bias which is plotted in Figure 7. From symmetry in the funnel plot, the studies are roughly symmetrically distributed on either side of the midline, indicating that a small sample effect is less likely to exist. There is insufficient evidence to support the publication bias.

### 3.2. The network pharmacology analysis of CPMs against AIC

#### 3.2.1. Effective interventions

We analyzed the characteristics of the 6 different DEX + CPMs interventions against AIC from the network meta-analysis. DEX + SM, DEX + SQFZ and DEX + AI + CI are the top three recommended interventions. Subsequently, the pharmacological mechanism of SM, SQFZ, and AI + CI against AIC was performed separately with the network pharmacology.

#### 3.2.2. Targets screen

**Figure 8:**
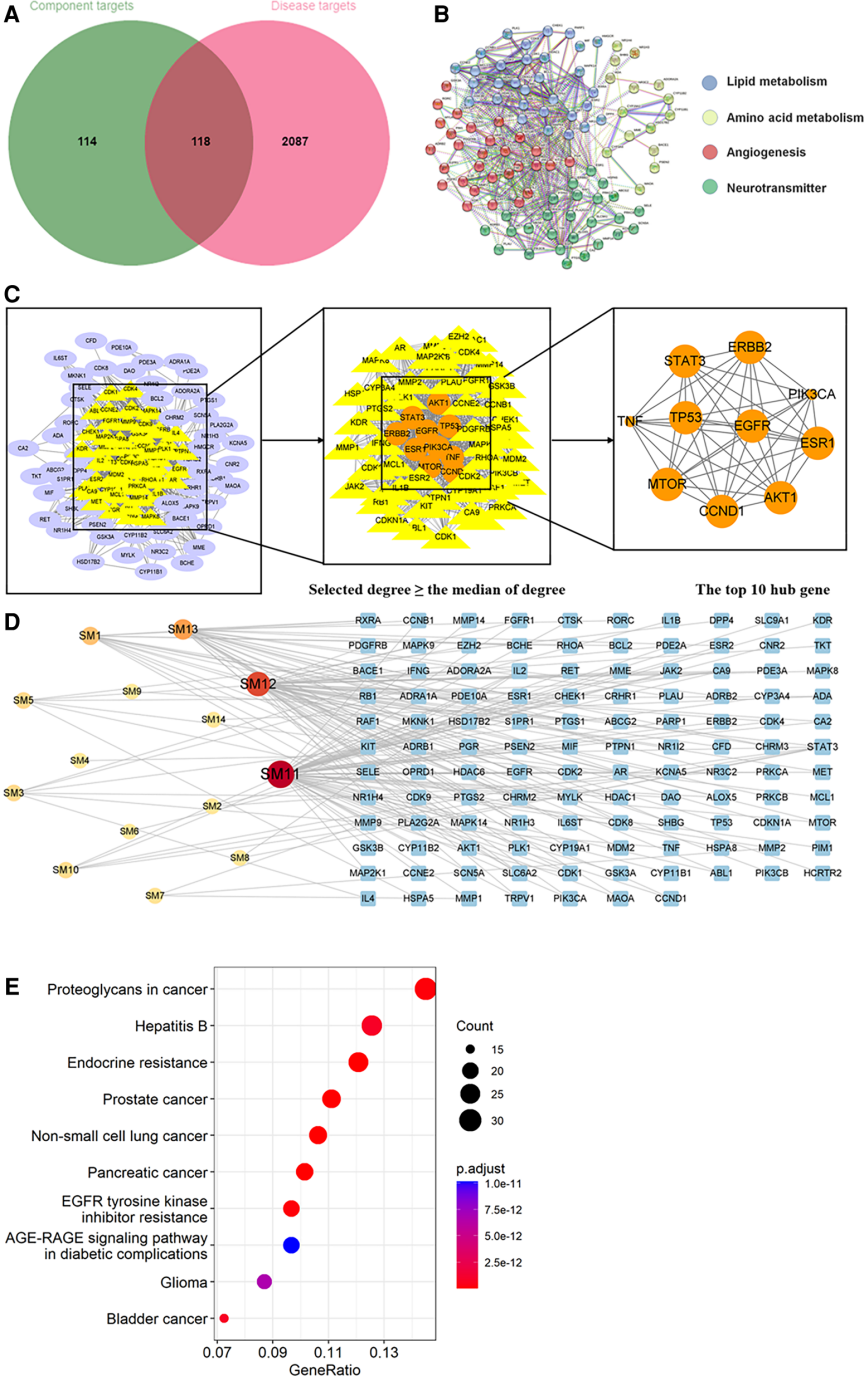
Analysis of target genes about shenmai injection. (**A**) Venn diagram of component target genes and disease target genes. The green circle stands for effective component target genes. The red circle stands for AIC target genes, and the intersection genes are shown in the middle. (**B**) The PPI network of the intersection genes. (**C**) Network topology analysis to screen the hub targets. (**D**) Component-Target Network of the active components and the target genes. (**E**) KEGG enrichment analysis of hub genes.

**Figure 9:**
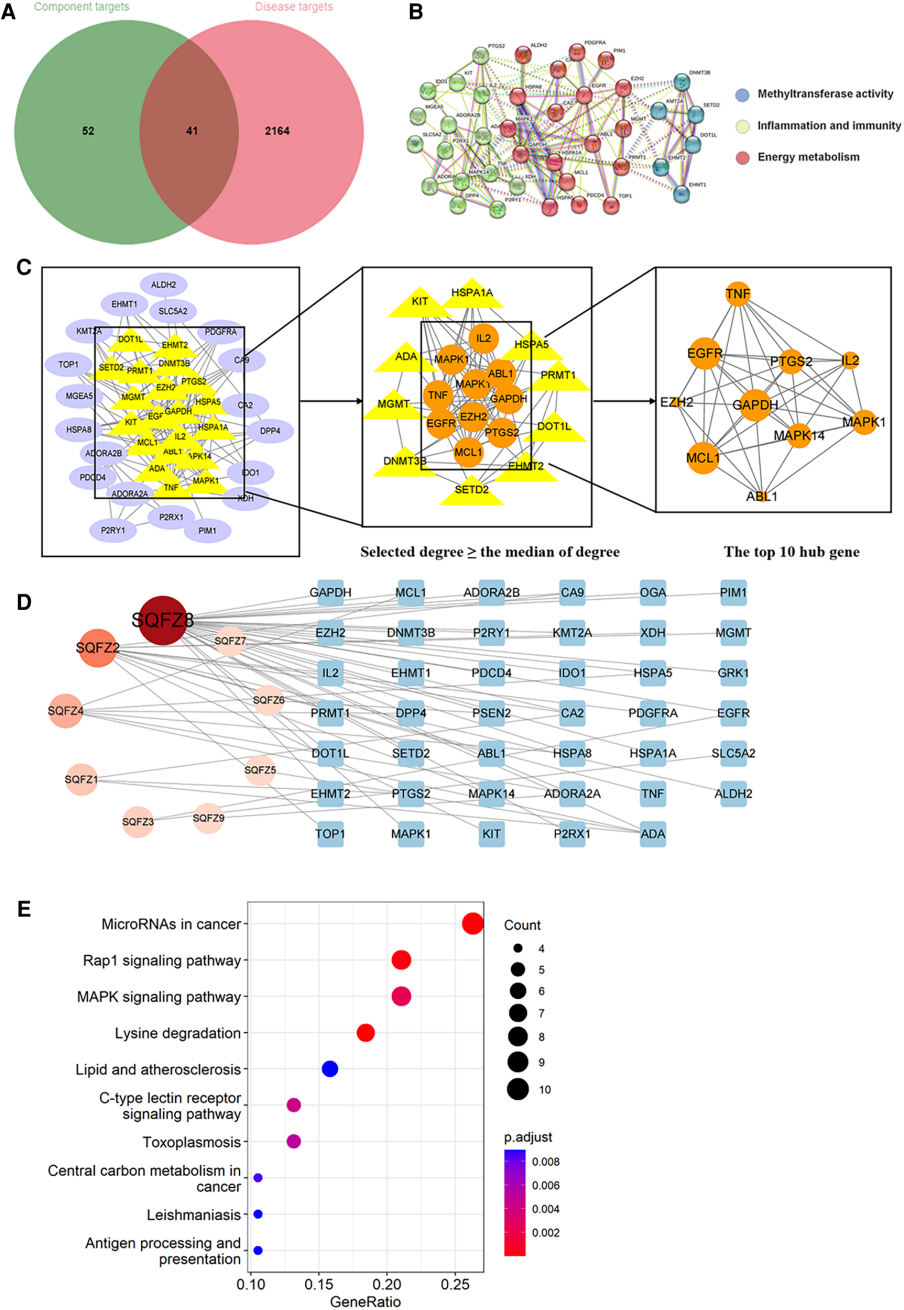
Analysis of target genes about shenqifuzheng injection. (**A**) Venn diagram of component target genes and disease target genes. The green circle stands for effective component target genes. The red circle stands for AIC target genes, and the intersection genes are shown in the middle. (**B**) The PPI network of the intersection genes. (**C**) Network topology analysis to screen the hub targets. (**D**) Component-Target Network of the active components and the target genes. (**E**) KEGG enrichment analysis of hub genes.

**Figure 10:**
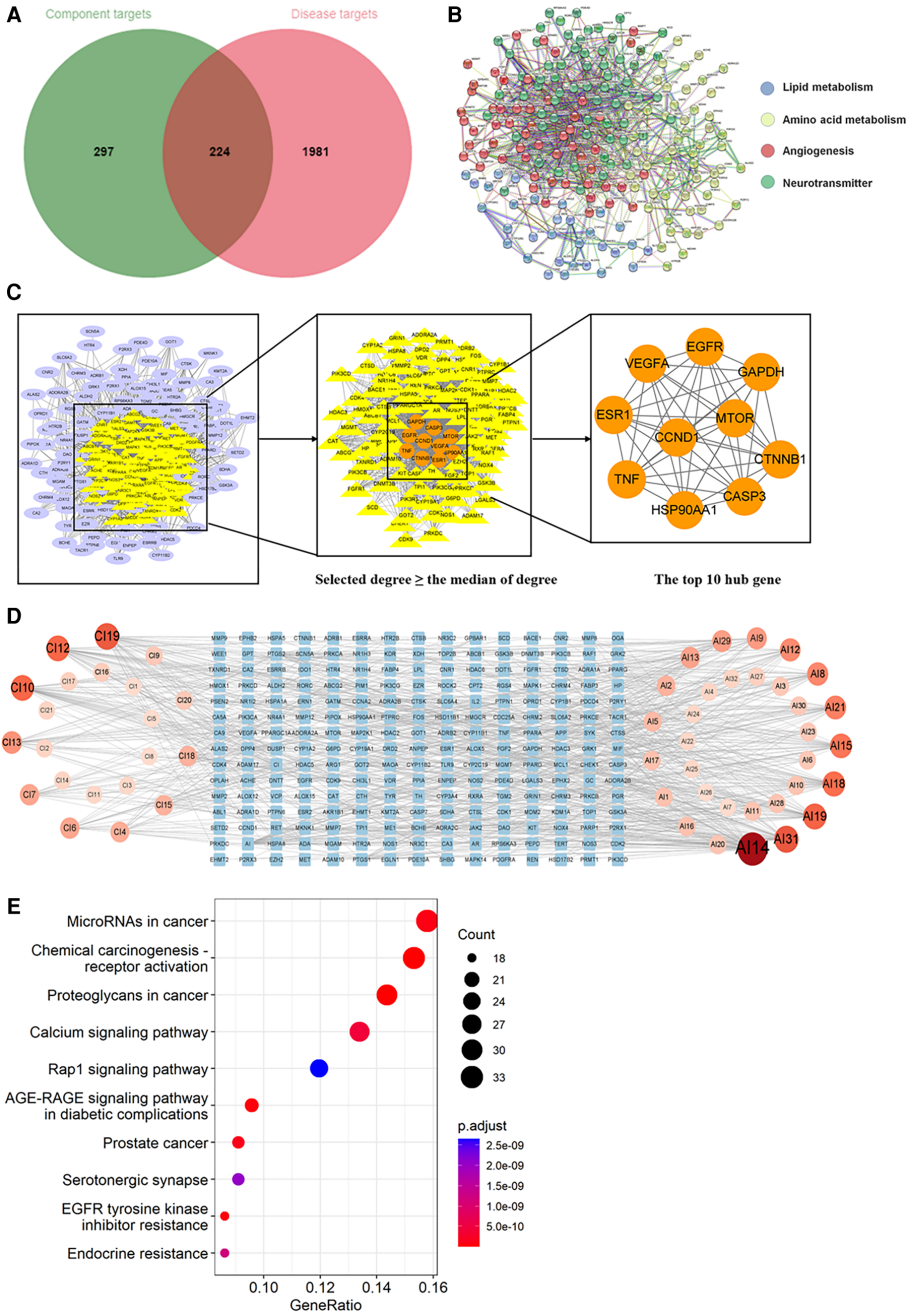
Analysis of target genes about Astragalus with cinobufotalin injection. (**A**) Venn diagram of component target genes and disease target genes. The green circle stands for effective component target genes. The red circle stands for AIC target genes, and the intersection genes are shown in the middle. (**B**) The PPI network of the intersection genes. (**C**) Network topology analysis to screen the hub targets. (**D**) Component-Target Network of the active components and the target genes. (**E**) KEGG enrichment analysis of hub genes.

Through searching the prescribed database, we screened out 2,205 AIC-related targets. For SM, 232 herb targets were obtained from reported literature, and 118 co-targets from herb and AIC were intersected in Figure 8A. For SQFZ, 93 herb targets were acquired, and 41 co-targets from herb and AIC were intersected in Figure 9A. As for AI + CI, 521 herb targets were received, and 224 co-targets from herb and AIC were crossed in Figure 10A.

#### 3.2.3. PPI network

As shown in Figures 8B, 9B, 10B, the co-targets of selected CPMs (SM, SQFZ, AI + CI) and AIC were respectively imported into the String platform. The PPI network of SM against AIC contains 118 nodes and 1,283 edges, and the median degree value screened out 60 core targets. Its top 10 hub genes are shown in Figure 8C: TP53, ESR1, EGFR, STAT3, AKT1, CCND1, MTOR, ERBB2, TNF, and PIK3CA. The PPI network of SQFZ against AIC contains 41 nodes and 147 edges, and the median degree value screened out 20 core targets. Its top 10 hub genes are shown in Figure 9C: GAPDH, EGFR, EZH2, MCL1, TNF, MAPK14, MAPK1, IL2, PTGS2 and ABL1. The PPI network of AI + CI contains 224 nodes and 2,908 edges, and the median degree value screened out 121 core targets. Its top 10 hub genes are shown in Figure 10C: GAPDH, EGFR, CASP3, TNF, CTNNB1, VEGFA, ESR1, HSP90AA1, MTOR and CCND1.

#### 3.2.4. Component-target network

As shown in Figures 8D, 9D, 10D, the core ingredients and targets of selected CPMs (SM, SQFZ, AI + CI) against AIC were imported into Cytoscape 3.8.2 to establish the component-target (C-T) network. The C-T network of SM against AIC contains 14 active compounds and 176 targets, and its active components include beta-elemene, uridine, stigmasterol, ruscogenin, Parisaponin I, orchinol, ophiopogonin D, ophiopogonanone E, n-trans-feruloyltyramine, Notoginsenoside R1, guanosine, ginsenoside Rg1, ginsenoside Rf, diosgenin. The C-T network of SQFZ against AIC contains 9 active compounds and 55 targets, and its active components include uridine, ononin, lobetyolin, guanosine, cytidine, astragaloside VI, astragaloside II, adenine and adenosine. The C-T network of AI + CI against AIC contains 53 active compounds and 651 targets, and its active components mainly include isoastragaloside II, adenosine, uridine, cycloastragenol, ethenzamide, cholesterol, formononetin, glycine, calycosin and phenylalanine.

#### 3.2.5. KEGG enrichment analysis

KEGG enrichment analysis was performed with the co-targets of selected CPMs (SM, SQFZ, AI + CI) and AIC respectively.

The KEGG pathway annotation indicated that 267 pathways were yielded. The top 10 KEGG pathways in Figure 8E except some abnormal transcription of cancers, were mainly including EGFR tyrosine kinase inhibitor resistance and AGE-RAGE signaling pathway in diabetic complications. And their detailed characteristics were listed in Supplementary Table S3.

The KEGG pathway annotation indicated that 205 pathways were yielded. The top 10 KEGG pathways in Figure 9E were mainly including lysine degradation, microRNAs in cancer, Rap1 signaling pathway, MAPK signaling pathway, C-type lectin receptor signaling pathway and lipid and atherosclerosis, and their characteristics were listed in Supplementary Table S4.

The KEGG pathway annotation indicated that 276 pathways were yielded. The top 10 KEGG pathways in Figure 10E were mainly including AGE-RAGE signaling pathway in diabetic complications, EGFR tyrosine kinase inhibitor resistance, MicroRNAs in cancer, calcium signaling pathway, and serotonergic synapse and Rap1 signaling pathway except some abnormal transcription of cancers, and their characteristics were listed in Supplementary Table S5.

## 4. Discussion

AIC clinically encompasses any cardiac complications containing early myocardial injury that can lead to acute or chronic left ventricular dysfunction and, subsequently, irreversible heart failure (44). From a contemporary cohort of 2,625 anthracycline-treated patients, AIC incidence was up to 9% after a median time of 5.2 years (45). Several studies have reported favorable advantages of combining CPMs and western medicine treatments in alleviating AIC (46, 47). To evaluate the efficacy of CPMs combined with DEX against AIC, and further explore their effective pharmacological mechanism, we adopted a combination of network meta-analysis and network pharmacological approach.

### 4.1. The network meta-analysis of the combination of CPMs and DEX against AIC

Network meta-analysis could compare multiple treatments simultaneously and consider other potential sources of heterogeneity (48), which could better identify the best approach for against AIC. According to the network meta-analysis results, all DEX + CPMs treatments could significantly relieve the AIC when compared with the DEX alone. The leading evaluating indicators include cTnI level, CK-MB level, LVEF value, and ECG abnormal rate. cTnI and CK-MB are the representative indexes of myocardial zymograms, and always as blood biomarkers identified to detect cardiac damage (49). The elevated levels of cTnI and CK-MB could reflect the changes in myocardial injury degree in different periods (50, 51). Our results revealed that five DEX + CPMs interventions (DEX + CI, DEX + SQFZ, DEX + SM, DEX + AI, and DEX + AI + CI) could significantly decrease the levels of cTnI and CK-MB. Among those effective interventions, DEX + SM treatment has higher advantages in lowering the cTnI level, and DEX + SQFZ treatment has higher advantages in reducing the CK-MB level. LVEF is the common index in assessing left ventricular systolic function and is usually employed in the early detection of cardiotoxicity (52). It is worth noting that DEX + SM treatment also has significant advantages over other therapies in improving LVEF value. ECG could reflect various states of the subject's heart, such as the origin of the rhythm, beat frequency, myocardial ischemia or not, and electrolyte changes in the body (53). The abnormal ECG related to suspicion of cardiotoxicity includes ST-T segment change, sinus tachycardia, atrioventricular block, and premature beat. Depression or elevations of the ST-T segment was the best indicator of myocardial blood supply, premature beat could trigger sustained arrhythmias and cause cardiomyopathies (54), and atrioventricular block and sinus tachycardia were also the most common myocarditis complication (55). Our results exhibited that DEX + WXKL treatment was the most effective in improving the ST-T segment, DEX + SM treatment was the best intervention in mitigating atrioventricular block, and DEX + AI + CI treatment was the best intervention in relieving both sinus tachycardia and premature beat.

In general, DEX + SM, DEX + SQFZ, and DEX + AI + CI were the top three recommended interventions against AIC from the network meta-analysis in the present study. Subsequently, we conducted network pharmacological analysis on the top three interventions to explore their potential mechanism of anti-AIC respectively.

### 4.2. The network pharmacology analysis of CPMs against AIC

Network pharmacology is a promising way to understand disease mechanisms and medication action mechanisms in the context of larger biological networks (56), which is conducive to exploring the target research of DEX + CPMs against AIC. However, DEX as a western medicine with a single component, which mechanism and targets have been clearly reported. That both chelate iron and target topoisomerase 2 alpha (top2*α*) (57). Therefore, the multi-component and multi- target of CMPs became the core content of our exploration under the network pharmacology.

SM, a commonly used CPMs for treating cardiovascular diseases in the clinic, consists of extractions from Red Ginseng (Chinese name: Hongshen) and Radix Ophiopogonis (Chinese name: Maidong) (58, 59). It was widely used in cardiovascular diseases and has a certain synergistic effect when combined with chemotherapy drugs, which could reduce the side effects caused by chemotherapy drugs. Some studies have shown that SM can reduce AIC by regulating inflammation and restoring cardiac dysfunction (60). The network pharmacological analysis collected 14 components and 176 potential targets of SM against AIC. Several identified components in SM have been reported with therapeutic effects on cardiac diseases, which supported our results of network prediction. For example, beta-elemene could alleviate heart failure by blocking lipid-induced inflammatory pathways (61). The cardiovascular protection effects of stigmasterol have been widely documented (62–64). Saponins, including ginsenosides, ophiopogonin, and diosgenin, are regarded as effective options for anti-AIC (65–67). From target prediction and pathway analysis, EGFR tyrosine kinase inhibitor resistance may play a vital role in the pharmacological mechanism of SM against AIC. As we all know, the abnormal cardiomyocyte homeostasis induced by autophagy is essential for AIC occurrence, and EGFR tyrosine kinase inhibitor has been reported could induce autophagy in cancer cells (68, 69). Meanwhile, literature reported that EGFR tyrosine kinase inhibitor could activate mitochondrial apoptosis, which is also a crucial factor for AIC (70). All evidence implied that the anti-AIC effect of SM may relate with inhibiting autophagy and mitochondrial apoptosis through regulating the EGFR tyrosine kinase inhibitor resistance pathway. In addition, the AGE-RAGE signaling pathway in diabetic complications may be another important pathway for SM against AIC from our results. Literature showed that AGE-RAGE signaling activation could stimulate inflammation response to exaggerate inflammatory cell influx into the susceptible myocardium (71), while targeting the AGE-RAGE pathway is a potential therapeutic strategy for ameliorating myocardial injury (72). And AGE-RAGE signaling cascade could result in numerous profibrotic growth factors secretion and collagen deposition, which may contribute to the left ventricle stiffness, one of the clinical manifestations of AIC (73, 74). Thus, the AGE-RAGE signaling pathway in diabetic complications might be the critical pathway of SM in improving anthracycline-induced left ventricular dysfunction.

SQFZ is concocted from Radix Astragali (Chinese name: Huangqi) and Radix Codonopsis (Chinese name: Dangshen), and is clinically suggested as a complementary treatment of chemotherapy, and clinical practices suggest that SQFZ has therapeutic effects on cardiovascular diseases (75, 76). Intensive researches reported that SQFZ exerts protective through improving myocardial energy metabolism, inhibiting cell adhesion and inflammatory reaction, and reducing myocardial apoptosis and avoiding ventricular remodel (77). The network pharmacological analysis collected 9 components and 55 potential targets of SQFZ against AIC. Among anti-AIC components of SQFZ, several members have been recognized by published studies. For instance, ononin, a natural isoflavone glycoside, could alleviate endoplasmic reticulum stress in AIC by modulating apoptosis-related signaling pathways (78). Astragaloside is reported as an active agent for AIC inhibition by inhibiting autophagy and oxidative stress (79–81). From target prediction and pathway analysis, the pharmacological mechanism of SQFZ against AIC may associate with microRNAs in cancer, Rap1 signaling pathway, MAPK signaling pathway and lipid and atherosclerosis. MicroRNAs play an essential role in gene regulation associated with cardiotoxicity-related cell death, apoptosis, and differentiation (82). Several miRNAs were identified as biomarkers of AIC, such as miR-1, miR-126, and miR-210 (83). Recently studies also indicated that the targeted therapy of microRNA could alleviate AIC (84, 85) Moreover, research shows that inhibition of the MAPK signaling pathway could effectively alleviate AIC by regulating apoptosis (86). And the lipid-lowering drugs were confirmed as promising cardioprotective agents in AIC patients (87). In the next step, experimental validation could be carried out for a more confirmed mechanism of SQFZ against AIC.

AI is the extraction of Radix Astragali, and is generally used in viral myocarditis, enteritis, and hepatitis (88). CI is the extraction of cinobufotalin (Chinese name: Huachansu), which is the major anti-tumor component isolated from toad venom and has been used clinically for various cancers (89). These were both seen to be commonly used CPMs in clinical practice, as complementary treatment to recommended Western therapies. AI could enhance myocardial contractility, improve circulation, protect myocardial cells and regulate immunity (90). CI has a shortening effect on action potential duration and an inhibitory effect on Na^+^, K ^(+)^-ATPase activity along with its cardiotonic effect (91). The network pharmacological analysis collected 53 components and 651 potential targets of AI + CI against AIC. The top 10 components identified from the C-T network of AI + CI against AIC have shown anti-cardiotoxicity activity in published studies, indicating their potentiality in anti-ACT (92). From target prediction and pathway analysis, there are several co-pathways of AI + CI, SM, and SQFZ in treating AIC. For the anti-AIC mechanism of AI + CI and SM, pathways of EGFR tyrosine kinase inhibitor resistance and the AGE-RAGE signaling pathway in diabetic complications both play important roles. As for the anti-AIC mechanism of AI + CI and SQFZ, microRNAs in cancer and the Rap1 signaling pathway were significantly enriched. It may relate to some same ingredients of herbs in these CPMs. Notably, the calcium signaling pathway is the unique enrichment pathway of AI + CI against AIC, compared with SM and SQFZ. Calcium ion overload is well known to be vital in the pathogenesis of heart dysfunctions, especially arrhythmia. The pathological cellular Ca^2+^ overload could lead to an accelerated beating rhythm in ventricular myocytes and caused arrhythmia (93). Moreover, the doxorubicin metabolite could target calsequestrin type 2, increasing cytoplasmic Ca^2+^ concentration, and triggering an arrhythmogenic state (94). These results might explain why AI + CI treatment has advantages in improving ECG abnormalities of cardiotoxic substances.

## 5. Limitations

Inevitably, there are some limitations in our study. Firstly, the effectiveness of DEX + CPMs treatments against AIC still needs further validation with more long-term and high-quality population samples. Secondly, it is inevitable to choose multiple-drug chemotherapy schemes considering the complex condition of tumor patients in clinically, RCTs that use only anthracycline chemotherapy regimens need to be included as much as possible in the future, in order to eliminate interference from other factors. Thirdly, included studies had inadequate descriptions of randomization, allocation concealment, and blinding method, which may lead to baseline wander. Finally, this study was based on data analysis only, and further *in vivo* and *in vitro* experiments are necessary to support our results.

## 6. Conclusion

We conducted a network meta-analysis integrated with network pharmacology analysis to compare the efficacy of different DEX + CPMs treatments against AIC, and explore their potential pharmacological mechanisms. Multifaceted evidence revealed that DEX + CPMs treatments have obvious advantages over DEX alone in anti-AIC. DEX + SM, DEX + SQFZ, and DEX + AI + CI might be the preferred intervention for improving LVEF value, CK-MB level, and ECG abnormalities. And network pharmacology discovered that CPMs has the characteristics of multiple pathways, multiple components, and multiple targets in alleviating AIC. Collectively, the present study will provide clinicians and researchers with detailed comparisons of therapeutic strategies and references.

## Data Availability

The original contributions presented in the study are included in the article/**Supplementary Material**, further inquiries can be directed to the corresponding author.
