# Supplementary material for: Comparative efficacy and pharmacological mechanism of Chinese patent medicines against anthracycline-induced cardiotoxicity: An integrated study of network meta-analysis and network pharmacology approach
Source: Front Cardiovasc Med. 2023 Apr 24;10:1126110. doi: 10.3389/fcvm.2023.1126110 (PMC10164985; doi:10.3389/fcvm.2023.1126110)
Supplement: Supplementary file 1 [file Table1.docx]

**Supplementary Captions**

Supplementary table 1. The detailed search terms, example search strategy (PubMed and CNKI).

Supplementary table 2. The detailed information of main CMPs.

Supplementary table 3. Characteristics of Top 10 KEGG Pathways of anti-AIC of Shenmai injection.

Supplementary table 4. Characteristics of Top 10 KEGG Pathways of anti-AIC of Shenqifuzheng injection.

Supplementary table 5. Characteristics of Top 10 KEGG Pathways of anti-AIC of Astragalus with Cinobufotalin injection.

Supplementary table 1. The detailed search terms, example search strategy (PubMed and CNKI).

**Example search strategy (PubMed)**: (“Anthracyclines” [Title/Abstract] or “Adriamycin” [Title/Abstract] or “Epirubicin” [Title/Abstract] or “Pirarubicin” [Title/Abstract] or “Daunorubicin” [Title/Abstract] or “Idarubicin” [Title/Abstract] or “Aclarubicin” [Title/Abstract] or “Daunomycin” [Title/Abstract] or “Carminomycin” [Title/Abstract] or “Mitoxantrone” [Title/Abstract] or “Liposome doxorubicin” [Title/Abstract] or “Doxorubicin” [Title/Abstract] or “Doxorubicin hydrochloride” [Title/Abstract] or “Aclacinomycin” [Title/Abstract] or “Aclacinon” [Title/Abstract] or “Nogalamycin” [Title/Abstract] or “Amrubicin” [Title/Abstract] or “Demethoxydaunor Ubicin” [Title/Abstract] or “ADM” [Title/Abstract] or “ACM” [Title/Abstract] or “DNR” [Title/Abstract] or “EPI” [Title/Abstract] or “THP” [Title/Abstract]) and (“Carvedilol” [Title/Abstract] or “Amifostine” [Title/Abstract] or “Dexrazoxane” [Title/Abstract] or “beta blockers” [Title/Abstract] or “Trimetazidine” [Title/Abstract] or “Candesartan” [Title/Abstract] or “Enalapril” [Title/Abstract] or “Metoprolol” [Title/Abstract] or “Shenfu Injection” [Title/Abstract] or “Xiangdan injection” [Title/Abstract] or “Shenqi Fuzheng Injection” [Title/Abstract] or “Shensong Yangxin Capsules” [Title/Abstract] or “Danshen Injection” [Title/Abstract] or “Fufang Kushen Injection” [Title/Abstract] or “Safflower yellow injection” [Title/Abstract] or “Honghua Injection” [Title/Abstract] or “Huachangsu Injection” [Title/Abstract] or “Astragalus polysaccharide injection” [Title/Abstract] or “APS injection” [Title/Abstract] or “Huangqi Injection” [Title/Abstract] or “Huangqi Shengmai Yin” [Title/Abstract] or “Qili Qiangxin capsule” [Title/Abstract] or “Sacubitril Valsartan Sodium Tablets” [Title/Abstract] or “Shexiang Baoxin Wan” [Title/Abstract] or “Shengmai Injection” [Title/Abstract] or “Wenxin Granules” [Title/Abstract] or “Xinmai long Injection” [Title/Abstract] or “Yixin Kangtai Capsules” [Title/Abstract] or “Ginkgo Leaf Extract and Dipyridamole Injection” [Title/Abstract] or “Ginkgo biloba Damo injection” [Title/Abstract] or “Elemene Injection” [Title/Abstract] or “coenzyme q10” [Title/Abstract] or “nebivolol” [Title/Abstract] or “spironolactone”)

**Example search strategy (CNKI)**: (SU %= 'Enhuan' OR SU %= 'Ameisu' OR SU %='Biaoameisu' OR SU %= 'Binanameisu' OR SU %= 'Rouhongmeisu ' OR SU %= 'Qujiayangrouhongmeisu ' OR SU %= 'Akelameisu ' OR SU %= 'Anlemeisu ' OR SU %= 'Daonuomeisu ' OR SU %= 'Hongbimeisu ' OR SU %= 'Roumaomeisu ' OR SU %= 'Qujiayangrouhongmeisu ' OR SU %= 'Yanghongmeisu ' OR SU %= 'Kaminuomeisu ' OR SU %= 'Mituoenkun ' OR SU %= 'Zhizhitiameisu ' OR SU %= 'Duoroubixing ' OR SU %= 'Biaorouobixing ' OR SU %= 'Biroubixing ' OR SU %= 'Aroubixing ' OR SU %= 'Yidabixing ' OR SU %= 'Nuolameisu ' OR SU %= 'Karoubixing ' OR SU %= 'Anroubixing ') AND (SU %= 'βsoutizuduanji ' OR SU %= 'Kaweidiluo ' OR SU %= 'Anlinting ' OR SU %= 'Youbingyaan ' OR SU %= 'Shenfu Zhusheye ' OR SU %= 'Shenlinggubenwan ' OR SU %= 'Shenmai ' OR SU %= 'Xiangdan Zhusheye ' OR SU %= ''Shenqifuzheng Zhusheye ' OR SU %= 'Shensong Yangxin Jiaonang ' OR SU %= 'Qumeitaqin ' OR SU %= 'Danshen Zhusheye ' OR SU %= 'Fufang Kuhen zhusheye ' OR SU %= 'Honghua Huangsesu zhusheye ' OR SU %= 'Honghua Zhusheye ' OR SU %= 'Huachansu Zhusheye ' OR SU %= 'Huangqiduotang Zhusheye ' OR SU %= 'Huangqi Zhusheye ' OR SU %= 'Qili Qiangxin jiaonang ' OR SU %= 'Shakubaquxieshatanna ' OR SU %= 'Shexiang baoxinwan ' OR SU %= 'Shengmai Zhusheye ' OR SU %= 'Wenxinkeli ' OR SU %= 'Xinmailong Zhusheye ' OR SU %= 'Yinapuli ' OR SU %= 'Meituoluoer ' OR SU %= 'Yixinkangtai Jiaonang ' OR SU %= 'Yinxing Damo Zhesheye ' OR SU %= 'Youleizuosheng ' OR SU %= ' Lɑnxiɑnɡxi Zhusheye ' OR SU %= 'Zuokaniding ' OR SU %= 'Fumei q10' OR SU %= 'Naibiluoer ' OR SU %= 'Luoneizhi ')

Supplementary table 2

Supplementary table 2. The detailed information of main CMPs.

| Drug | China SFDA approval number | Main composition |
| --- | --- | --- |
| Wenxinkeli | Z10950026 | Radix Codonopsis, Polygonatum sibiricum, Panax Notoginseng, Amber, Nardostachys chinensis (Chinese name: Dangshen, Huangjing, Sanqi, Hupo, Gansong) |
| Shenqifuzheng injection | Z19990065 | Radix Codonopsis, Radix Astragali (Chinese name: Dangshen, Huangqi) |
| Astragalus injection | Z13020999 | Radix Astragali (Chinese name: Huangqi) |
| Shenmai injection | Z20093648 | Red Ginseng, Radix Ophiopogonis (Chinese name: Hongshen, Maidong) |
| Cinobufotalin injection | Z34020273 | Cinobufotalin (Chinese name: Huachansu) |

Supplementary table 3

Supplementary table 3. Characteristics of Top 10 KEGG Pathways of anti-AIC of Shenmai injection

| **ID** | **Description** | **P-Value** | **Count** |
| --- | --- | --- | --- |
| hsa01522 | Endocrine resistance | 7.40E-19 | 25 |
| hsa05223 | Non-small cell lung cancer | 1.39E-18 | 22 |
| hsa05212 | Pancreatic cancer | 8.85E-17 | 21 |
| hsa05215 | Prostate cancer | 1.17E-16 | 23 |
| hsa05205 | Proteoglycans in cancer | 2.99E-15 | 30 |
| hsa01521 | EGFR tyrosine kinase inhibitor resistance | 3.22E-15 | 20 |
| hsa05219 | Bladder cancer | 2.34E-14 | 15 |
| hsa05161 | Hepatitis B | 2.58E-14 | 26 |
| hsa05214 | Glioma | 2.27E-13 | 18 |
| hsa04933 | AGE-RAGE signaling pathway in diabetic complications | 4.09E-13 | 20 |

Supplementary table 4

Supplementary table 4. Characteristics of Top 10 KEGG Pathways of anti-AIC of Shenqifuzheng injection.

| **ID** | **Description** | **P-Value** | **Count** |
| --- | --- | --- | --- |
| hsa00310 | Lysine degradation | 1.18E-08 | 7 |
| hsa05206 | MicroRNAs in cancer | 9.60E-07 | 10 |
| hsa04015 | Rap1 signaling pathway | 4.12E-06 | 8 |
| hsa04010 | MAPK signaling pathway | 4.79E-05 | 8 |
| hsa04625 | C-type lectin receptor signaling pathway | 1.08E-04 | 5 |
| hsa05145 | Toxoplasmosis | 1.53E-04 | 5 |
| hsa05230 | Central carbon metabolism in cancer | 2.90E-04 | 4 |
| hsa05417 | Lipid and atherosclerosis | 4.17E-04 | 6 |
| hsa05140 | Leishmaniasis | 4.18E-04 | 4 |
| hsa04612 | Antigen processing and presentation | 4.39E-04 | 4 |

Supplementary table 5

Supplementary table 5. Characteristics of Top 10 KEGG Pathways of anti-AIC of Astragalus with Cinobufotalin injection.

| **ID** | **Description** | **P-Value** | **Count** |
| --- | --- | --- | --- |
| hsa05207 | Chemical carcinogenesis - receptor activation | 1.71E-16 | 32 |
| hsa05205 | Proteoglycans in cancer | 3.92E-15 | 30 |
| hsa04933 | AGE-RAGE signaling pathway in diabetic complications | 4.92E-13 | 20 |
| hsa01521 | EGFR tyrosine kinase inhibitor resistance | 7.07E-13 | 18 |
| hsa05206 | MicroRNAs in cancer | 1.67E-12 | 33 |
| hsa05215 | Prostate cancer | 2.87E-12 | 19 |
| hsa04020 | Calcium signaling pathway | 1.06E-11 | 28 |
| hsa01522 | Endocrine resistance | 3.45E-11 | 18 |
| hsa04726 | Serotonergic synapse | 6.65E-11 | 19 |
| hsa04015 | Rap1 signaling pathway | 9.60E-11 | 25 |
